# Association of inflammatory biomarkers with overall survival in burn patients: a systematic review and meta-analysis

**DOI:** 10.1186/s12873-024-00988-x

**Published:** 2024-04-29

**Authors:** Shima Nourigheimasi, Erfan Yazdani, Arshin Ghaedi, Monireh Khanzadeh, Brandon Lucke-Wold, Emma Dioso, Aida Bazrgar, Mehrnoosh Ebadi, Shokoufeh Khanzadeh

**Affiliations:** 1 School of Medicine, Arak University of Medical Sciences, Arak, Iran; 2 Medical School, Islamic Azad University, Tonekabon Branch, Tonekabon, Iran; 3 Student Research Committee, School of Medicine, Shiraz University of Medical Sciences, Shiraz, Iran; 4 Trauma Research Center, Shahid Rajaee (Emtiaz) Trauma Hospital, Shiraz University of Medical Sciences, Shiraz, Iran; 5 Geriatric & Gerontology Department, Medical School, Tehran University of Medical and Health Sciences, Tehran, Iran; 6 Department of Neurosurgery, University of Florida, Gainesville, USA; 7 University of Utah, Utah, USA; 8 Arak University of Medical Sciences, Arak, Iran; 9 Tabriz University of Medical Sciences, Tabriz, Iran

**Keywords:** Burn, Prognosis, Biomarker, Inflammation, Meta-analysis

## Abstract

**Introduction:**

The inflammatory response to burn injuries can lead to organ dysfunction that ultimately results in increased mortality and morbidity. This meta-analysis was conducted to determine the efficacy of inflammatory biomarkers, including the neutrophil to lymphocyte ratio (NLR), platelet to lymphocyte ratio (PLR), procalcitonin (PCT), and C-reactive protein (CRP) as predictive tools of mortality among burn patients.

**Material and methods:**

The biomarker levels of survivors and non-survivors were consolidated according to guidelines for Preferred Reporting Items for Systematic Reviews and Meta-Analyses (PRISMA). Three main databases were searched electronically: PubMed, Web of Science, and Scopus, on December 8, 2022. The Newcastle–Ottawa Quality Assessment Scale (NOS) was used to evaluate and score the methodological quality of the included studies. The standard mean difference (SMD) with a 95% confidence interval (CI) was utilized.

**Results:**

Twenty-four studies were included in our systematic review and meta-analysis, (3636 total burn patients), of whom 2878 survived. We found that deceased burn patients had elevated levels of NLR (SMD = 0.60, 95% CI; 0.19–1.00, *P* < 0.001), CRP (SMD = 0.80, 95% CI; 0.02–1.58, *P* = 0.04), and PCT (SMD = 0.85, 95% CI; 0.45–1.24, *P* < 0.001), compared to survivors. However, we found no association between PLR and mortality among burn patients (SMD = 0.00, 95% CI; -0.14–0.15, *P* < 0.001). In addition, CRP was significantly higher in non-survivors (SMD = 0.80, 95% CI; 0.02–1.58, P =0.04). Similar results were also found about PCT (SMD = 0.85, 95% CI; 0.45–1.24, P < 0.001). When we analyzed the PCT data, collected in the first 24-48 hours, we found similar results; the PCT level was significantly higher in non-survivors in the immediate postinjury-period (SMD = 0.67, 95% CI; 0.31–1.02, P < 0.001). There was no publication bias among studies on the role of NLR in burn (Egger’s test *P* = 0.91). The based cut-off values for NLR (13), CRP (71), and PCT (1.77) yielded sensitivities of 69.2%, 100%, and 93.33%, and specificities of 76%, 72.22%, and 72.22% respectively.

**Discussion/Conclusions:**

PCT is a marker of sepsis, therefore its elevated level is presumably associated with a higher incidence and severity of sepsis among non-survivors. In addition, NLR and CRP are promising biomarkers for predicting and guiding prevention against burn deaths in clinical settings.

## Introduction

The World Health Organization estimates that nearly 180,000 deaths are attributed to burns yearly. Burn injuries can worsen quickly, within days, as cardiogenic compromise and shock develop [1]. Subsequently, mortality associated with burn injuries is mainly due to infections contributing to sepsis, septic shock, and multiple organ dysfunction syndromes (MODS) [2]. Severe burn injuries cause a marked inflammatory stress response. As the inflammatory state progresses, pro-inflammatory cytokines are continuously released. In a state of severe or uncontrolled innate immune function, this activation can result in tissue injury and subsequent multi-organ failure. Anti-inflammatory cytokines are released simultaneously to dampen the response, but ultimately results in lymphocyte apoptosis and immunosuppression. The neutrophil-to-lymphocyte ratio (NLR) quantifies this balance [3].

The NLR is an inexpensive and straightforward clinical marker of infection [4]. The NLR uses complete blood count values to indicate systemic inflammation and can be calculated from absolute and relative levels [5]. Pathologically, blood neutrophils increase in response to an inflammatory process. In certain conditions, i.e., cachexia, blood neutrophil counts are note elevated, and a “false negative” occurs [6]. Similarly, lymphocyte counts indicate a patient’s immune status and typically decrease as the inflammatory response progresses. This decrease is notably delayed and may not be indicative of disease progression as previously indicated [7]. Recent cardiovascular and sepsis literature has suggested that the NLR is a more robust indicator of patient outcome versus neutrophil or lymphocyte counts alone [8, 9]. The NLR increases as the disease progresses, notably in inflammatory processes, and can serve as a prognostic factor in the risk of developing complications [10]. In their meta-analysis, Huang and colleagues found NLR to be a helpful biomarker in the prognosis of sepsis patients, explicitly noting that a higher NLR may correlate with a worse prognosis [6]. Similarly, Dragoescu and colleagues found a positive correlation between the NLR and prognosis of septic patients, with a notable increase for patients in septic shock [5].

C-reactive protein (CRP) is another biomarker that dramatically increases from injury, infection, and inflammation [11]. Specifically, CRP, an acute-phase protein, is closely associated with systemic inflammation, as CRP binds to the damaged cell membranes and contributes to the associated inflammatory response [12, 13]. Some studies have identified elevated CRP levels as a risk factor for developing sepsis [14–16]. Other studies suggest CRP is a confounding factor in identifying sepsis in burn patients as the chronic inflammatory process is a normal response to burn trauma [17].

Procalcitonin (PCT) is yet another biomarker valuable in the sepsis diagnosis [18]. Notably, PCT has been suggested to maintain high sensitivity and specificity when diagnosing post-burn sepsis during the middle and late stages of treating the injury [19–22]. Most importantly, PCT may have a role in guiding treatment and predicting the prognosis of sepsis in burn patients [23]. Controversy does exist within the literature, as false PCT elevations may occur in the early stages of the post-traumatic stage, given that PCT is also influenced by bacterial infections. PCT can also be influenced by non-infectious factors (i.e., stress and post-traumatic systemic inflammatory response syndrome) [24–27]. In 2021, Xu and colleagues retrospectively analyzed a large patient cohort with extensive burns and found that elevated PCT during the early phase can predict sepsis within 60 days of injury [28].

Finally, the platelet-to-lymphocyte ratio (PLR) helps indicate a shift in platelet and lymphocyte counts in acute inflammation. Many studies have evaluated the use of PLR in rheumatoid arthritis [29–31], cardiovascular disease [27, 32–34], and systemic lupus erythematosus [35–37]. Many studies have identified PLR as a prognostic indicator for early sepsis at presentation in the emergency department [38–42]. Angulo and colleagues evaluated PLR in burn patients; they found PLR to be reduced in patients who did not survive, and their data suggests PLR may help identify mortality in these patients [43].

With the increase in data regarding prognostic biomarkers and their role in inflammatory processes, a systemic review of these data in predicting outcomes in burn patients is necessary to support clinical decision-making [43–66]. Understanding the pathophysiology behind these biomarkers, could result in earlier intervention and improved outcomes among burn patients. This systemic review and meta-analysis aimed to compare the levels of inflammatory biomarkers (NLR, PLR, CRP, and PCT) between survivor and non-survivor burn patients to determine their efficacy as a prognostic biomarker for mortality and aid in the clinical management of burn patients.

## Material and method

This study was conducted according to Preferred Reporting Items for Systematic Reviews and Meta-Analyses (PRISMA).

### Data sources and searches

Using PubMed, Web of Science, Scopus, Cochrane lib., ScienceDirect, and Embase, an electronic search was conducted on December 8, 2022. The search terms included (((Neutrophil to lymphocyte ratio) OR NLR) OR (procalcitonin OR PCT) OR ((C-reactive protein) OR CRP) OR ((platelet to lymphocyte ratio) OR PLR)) AND (Burn*) AND (mortality OR prognosis OR outcome OR surviv*). Reference lists of the retrieved articles were investigated to find further relevant studies.

### Study selection

The inclusion criteria were as follows:

(i) studies on burn injuries assessing the prognostic role of the inflammatory biomarkers;
(ii) Availability of a mean and standard deviation (SD) of inflammatory biomarkers (interquartile range (IQR)) or median (range) from which the mean and standard were calculated;
(iii) Articles published in peer-reviewed journals.

The exclusion criteria were as follows:

(i) studies involved animals, cell lines, or human xenograft experiments;
(ii) case series, case reports, or review articles;
(iii) duplicate publications;
(iv) studies reporting odds ratio (OR), hazard ratio (HR), or risk ratio (RR) instead of mean and standard deviation (SD).

Two reviewers independently reviewed all the articles found through the search strategy. Disagreements were resolved by consensus. All potentially relevant papers were retrieved and evaluated for eligibility after excluding duplicate and obvious irrelevant articles. A corresponding author was contacted if any data was unclear or missing.

### Endpoint of interest

Survival prediction based on inflammatory biomarkers was the outcome of interest. We compared NLR, PLR, CRP, and PCT levels in the survivor verse non-survivor burn patients.

### Data extraction

Two authors independently collected data using predesigned abstraction forms. Disagreements were settled by consensus. Data extracted include the first author's name, year of publication, study location, study design (prospective or retrospective), number of survivors and non-survivors, and their biomarker levels (NLR, PLR, CRP, or PCT).

### Quality assessment

Studies were evaluated and scored according to the Newcastle–Ottawa Quality Assessment Scale (NOS), which consists of three sections: selection, comparability, and outcome. Scores of 6 or higher indicate high-quality studies.

### Statistical analyses

The statistical analysis was performed using STATA version 12.0 (Stata Corporation, College Station, TX, USA). The standard mean difference (SMD) with a 95% confidence interval (CI) was used, and subgroup analyses were also conducted based on study design (retrospective, prospective). Our meta-analysis used a random-effects model due to significant heterogeneity between studies. We assessed statistical heterogeneity using I^2^ statistics and Cochran's Q test. We used the method introduced by Wan et al. to estimate the mean and SD from the median (IQR and range) [67]. Publication bias was determined using visual inspection of funnel plots. The best cut-off value for each biomarker was defined as the highest value of sensitivity + specificity. Statistical significance was conceived as *p* < 0.05, and all statistical tests were two-sided.

## Results

### Eligible studies

**Fig. 1:**
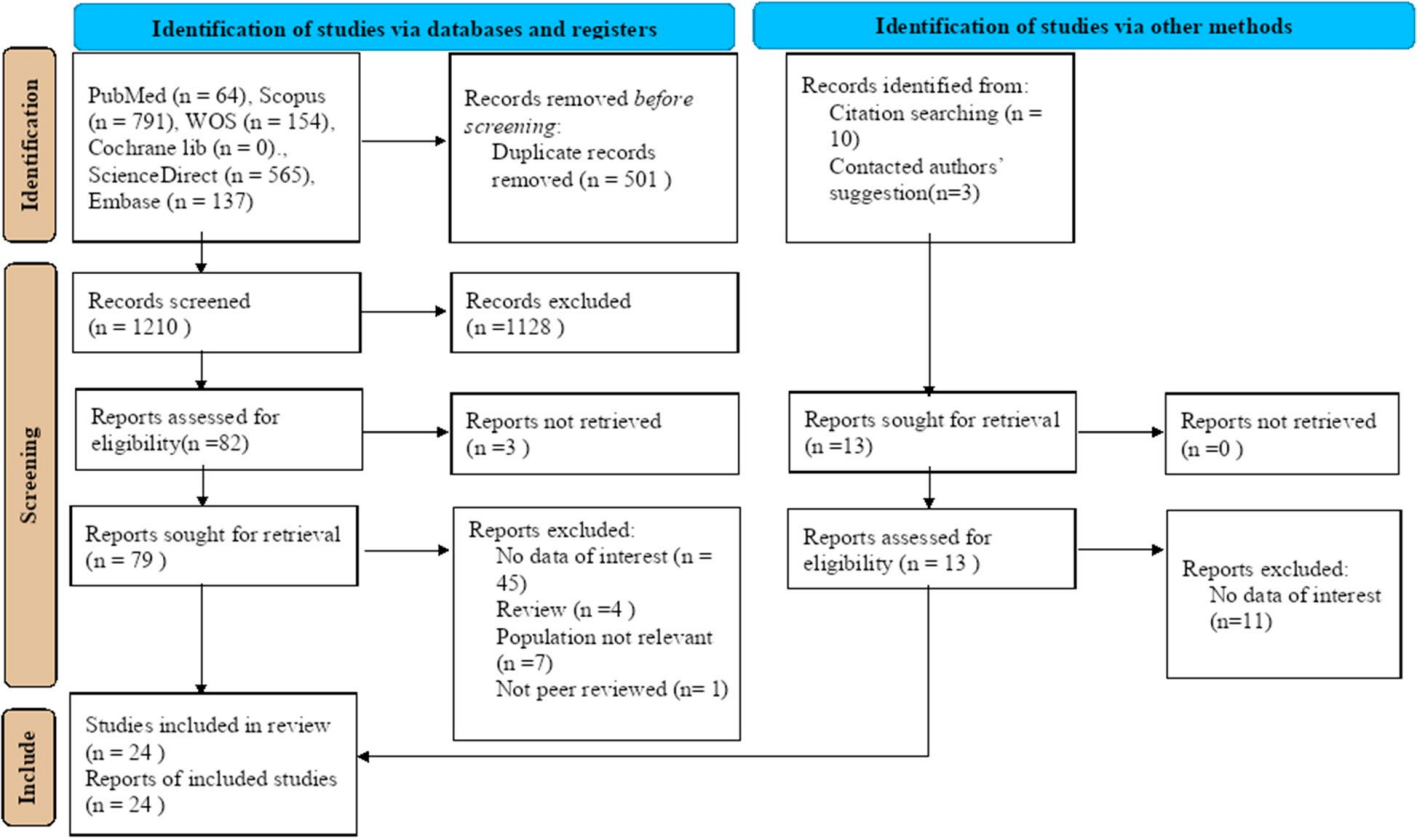
PRISMA 2020 Flow diagram for new systematic reviews, which includes searches of databases, registers, and other sources

A total of 1724 records were retrieved in the database search and manual search of the citation list of articles. After excluding duplicates and irrelevant documents, 24 studies were included in the systematic review and meta-analysis for a total of 3636 burn patients, of whom 2878 survived [43–66]. The process of inclusion and exclusion is detailed in the PRISMA flow diagram in Fig. 1.

### Study characteristics and quality assessment

Among the 24 included studies [43–66], 16 had retrospective designs [43–46, 48–50, 53, 56, 59, 60, 62–66]. Studies were conducted in Turkey(*n* = 5) [45, 49, 50, 62, 64], South Korea(*n* = 2) [58, 60], Portugal(*n* = 1) [53], Iraq(*n* = 2) [52, 57] Uruguay(*n* = 1) [43], India(*n* = 2) [44, 63], Indonesia(*n* = 1) [47], China(*n* = 5) [46, 56, 59, 65, 66], USA (*n* = 1) [55], France(*n* = 1) [54], Italy (*n* = 1) [61], Germany (*n* = 1) [51], and Sweden(*n* = 1) [48]. Ten studies reported in-hospital mortality [43–45, 47, 51, 54, 59, 62–64], three reported 90- day mortality [46, 56, 60], one reported 51-day mortality [65] and one reported 28-day mortality [66]. Other studies did not report any data regarding mortality [48, 49]. Quality of the studies was high, with scores ranging from 5 to 8. Table 1 lists the general characteristics of each study, and Table 2 shows the biomarker level including sensitivity and specificity for each study.

**Table 1:** General characteristics of included studies

Author	Year	Country	Design	Burn severity	Mortality time	Blood collection time
Gottschlich [55]	1992	USA	P	Mean TBSA:44.7%	Not declared	Between post-burn day 7 and 10
Carsin [54]	1997	France	P	TBSA > 30%	During hospitalization	After informed consent
Pileri [61]	2009	Italy	P	TBSA:10–18%	Not declared	On day 3
Altrichter [51]	2010	Germany	P	Severe burn injury with mean TBSA of 41.5%	During hospitalization	On day 3
Kim [58]	2012	South Korea	P	Mean TBSA:40%	Not declared	Not declared
Zu [66]	2015	China	R	APACHE Score: 22.94; SOFA Score: 7.96	28th day of treatment	During admission to the hospital
Piroglu [62]	2016	Turkey	R	Mean APACHE II:26.03	During ICU care except for first 48 h	First 48 h after admission to the hospital
Al-Ubadi [52]	2017	Iraq	P	TBSA: 10–95%	Not declared	Less than 6 h after admission to the hospital
Tiryaki [64]	2017	Turkey	R	TBSA:40.75%	During hospitalization	Not declared
Jasem [57]	2017	Iraq	P	TBSA: 10–95%	Not declared	During admission to the hospital
Cabral [53]	2018	Portugal	R	TBSA > 15%	Not declared	During admission to the hospital
Xu [65]	2018	China	R	TBSA > 30%	51-day mortality	During admission to the hospital
Cifciti [45]	2019	Turkey	R	Severe burns	During hospitalization	During admission to the hospital
Angulo [43]	2020	Uruguay	R	TBSA: 14 [7–23] %; ABSI: 6 [4–8]	During hospitalization	During admission to the hospital
Bhuyan [44]	2020	India	R	Not declared	During hospitalization	During admission to the hospital
O.N [47]	2020	Indonesia	P	Severe burns with TBSA of more than 10%	During hospitalization	During admission to the hospital
Temiz [49]	2020	Turkey	R	Severe burns with BSA of 15% and over	Not declared	Not declared
Akin [50]	2021	Turkey	R	Mean TBSA:40.73%	Not declared	During admission to the hospital
Qiu [46]	2021	China	R	Severe burns with TBSA of more than 30%	90-day mortality	On day 3
Sinha [63]	2021	India	R	Thermal burn injury with TBSA of 20 to 60%, without inhalation injury	During hospitalization	During admission to the hospital
Steinvall [48]	2021	Sweden	R	Severe burns	Not declared	During admission to the hospital
He [56]	2022	China	R	TBSA > 20%	90-day mortality	On the day before surgery
Liu [59]	2022	China	R	TBSA > 50%	During hospitalization	Within 48 h after injury
Park [60]	2022	South Korea	R	TBSA > 20%	90-day mortality	On the day before surgery

*TBSA* Total body surface area burned, *ABSI* Abbreviated burn severity index, *SOFA* Sequential organ failure assessment, *APACHE* Acute physiology and chronic health evaluation, *P* Prospective, *R* Retrospective

**Table 2:** The biomarker level and its sensitivity and specificity in each study

Author	Biomarker	Survivor		Non-survivor		Cut-off	Sensitivity	specificity	NOS score
		Number	Biomarker level	Number	Biomarker level				
Gottschlich [55]	CRP	47	11.40 ± 0.70	13	19.90 ± 1.60	15	77	79	8
Carsin [54]	PCT	21	7617 ± 14271	11	17733 ± 35626.08	_	_ _	_	7
Pileri [61]	CRP	16	400.00 ± 80.00	5	252.0 ± 125.00	_	_	_	7
Altrichter [51]	PCT	25	2.97 ± 1.80	7	5.77 ± 1.10	0.61	100	59	7
Kim [58]	PCT	117	6.81 ± 5.33	58	48.74 ± 40.09	2	77.6	82.1	6
Zu [66]	CRP	79	16.37 ± 4.06	19	18.21 ± 4.83	_	_	_	7
	PCT	79	59.62 ± 10.65	19	64.62 ± 19.37	_	_	_	
Piroglu [62]	CRP	40	106.58 ± 60.70	30	77.28 ± 82.31	_	_	_	8
	PCT	40	0.90 ± 0.98	30	16.73 ± 28.86	2	86.67	70	
Al-Ubadi [52]	CRP	35	20.60 ± 26.10	15	25.40 ± 32.45	_	_	_	7
	PCT		827.0 ± 852.00		773.0 ± 799.00	_	_	_	
Tiryaki [64]	CRP	106	75.88 ± 62.40	9	72.10 ± 70.73	_	_ _	_	6
Jasem [57]	PCT	35	827.0 ± 852.00	15	773 ± 799	_	_ _	_	8
Cabral [53]	PCT	68	2.04 ± 3.87	33	7.00 ± 6.06	_	_	_	8
Xu [65]	CRP	16	25.60 ± 3.57	22	23.93 ± 1.26	_	_	_	8
	PCT	16	2.43 ± 2.51	22	5.10 ± 5.07	24	75	69.7	
Cifciti [45]	NLR	314	5.54 ± 5.65	52	10.94 ± 7.63	_	_	_	6
Angulo [43]	NLR	75	8.63 ± 5.74	13	16.80 ± 13.28	13	69.2	76	7
	PLR		115.33 ± 57.90	13	82.23 ± 87.79	60	54.5	95.8	
Bhuyan [44]	NLR	194	7.23 ± 3.25	48	14.44 ± 6.95	_	_	_	6
O.N [47]	NLR	15	8.79 ± 5.04	3	12.61 ± 7.77	_	_	_	5
	CRP	15	15.78 ± 7.57	3	21.50 ± 2.50	_	_	_	
Temiz [49]	NLR	109	6.34 ± 12.13	24	12.96 ± 9.70	_	_	_	5
	CRP	109	24.76 ± 49.81	24	39.35 ± 56.73	_	_	_	
	PLR	109	82.77 ± 94.30	24	46.56 ± 31.34	_	_	_	
Akin [50]	CRP	109	60.03 ± 60.23	24	161.4 ± 99.00	185.5	_	_	6
Qiu [46]	NLR	522	10.55 ± 7.45	55	11.62 ± 6.61	10.5	60	70.10	7
Sinha [63]	CRP	36	56.92 ± 24.38	15	91.33 ± 7.09	71	100	72.22	7
	PCT	36	1377 ± 1015.1	15	2673.9 ± 1505.45	1.772	93.33	72.22	
Steinvall [48]	NLR	185	9.53 ± 7.08	37	12.91 ± 8.62	_	_	_	8
He [56]	NLR	133	9.30 ± 5.24	36	8.96 ± 3.62	_	_	_	7
Liu [59]	PCT	112	2.14 ± 2.76	27	12.70 ± 15.16	5.4	70.4	81.3	7
Park [60]	NLR	533	10.60 ± 19.10	198	11.20 ± 15.70	_	_	_	8
	PLR	533	276.00 ± 464.00	198	304.00 ± 606.00	_	_	_	

*NLR* Neutrophil to lymphocyte ratio, *CRP* C-reactive protein, *PCT* Procalcitonin, *PLR* Platelet to lymphocyte ratio, *NOS* Newcastle–Ottawa Quality Assessment Scale

### Comparison of NLR between survivors and non-survivors

Five studies assessed the NLR level of patients at admission to the hospital [43–45, 47, 48], two studies assessed it on the day before surgery [56, 60], one study reported it on day 3 [46], and one study did not report the data [49] (Tables 1 and 2).

**Fig. 2:**
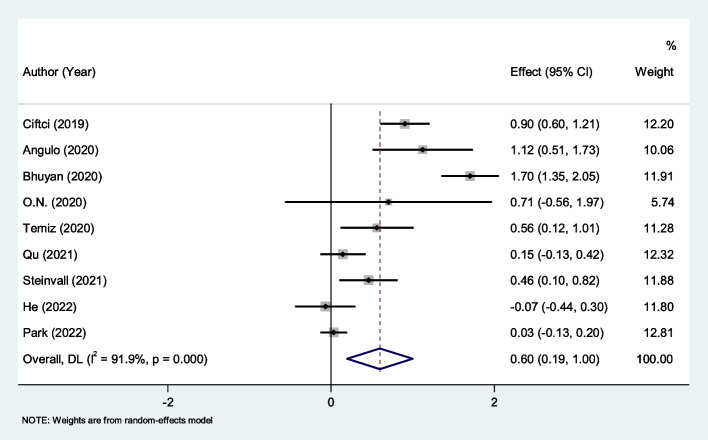
Meta-analysis of differences in NLR level between survivor and non-survivor burned patients

After pooling the data of nine studies with 2546 burned patients, including 2080 survivors [43–49], NLR was significantly higher in non-survivors (SMD = 0.60, 95% CI; 0.19–1.00, *P* < 0.001). The results of the studies showed significant heterogeneity (I^2^ = 91.9%, *P* < 0.001; Fig. 2). We therefore used the random effect model in our meta-analysis.

**Fig. 3:**
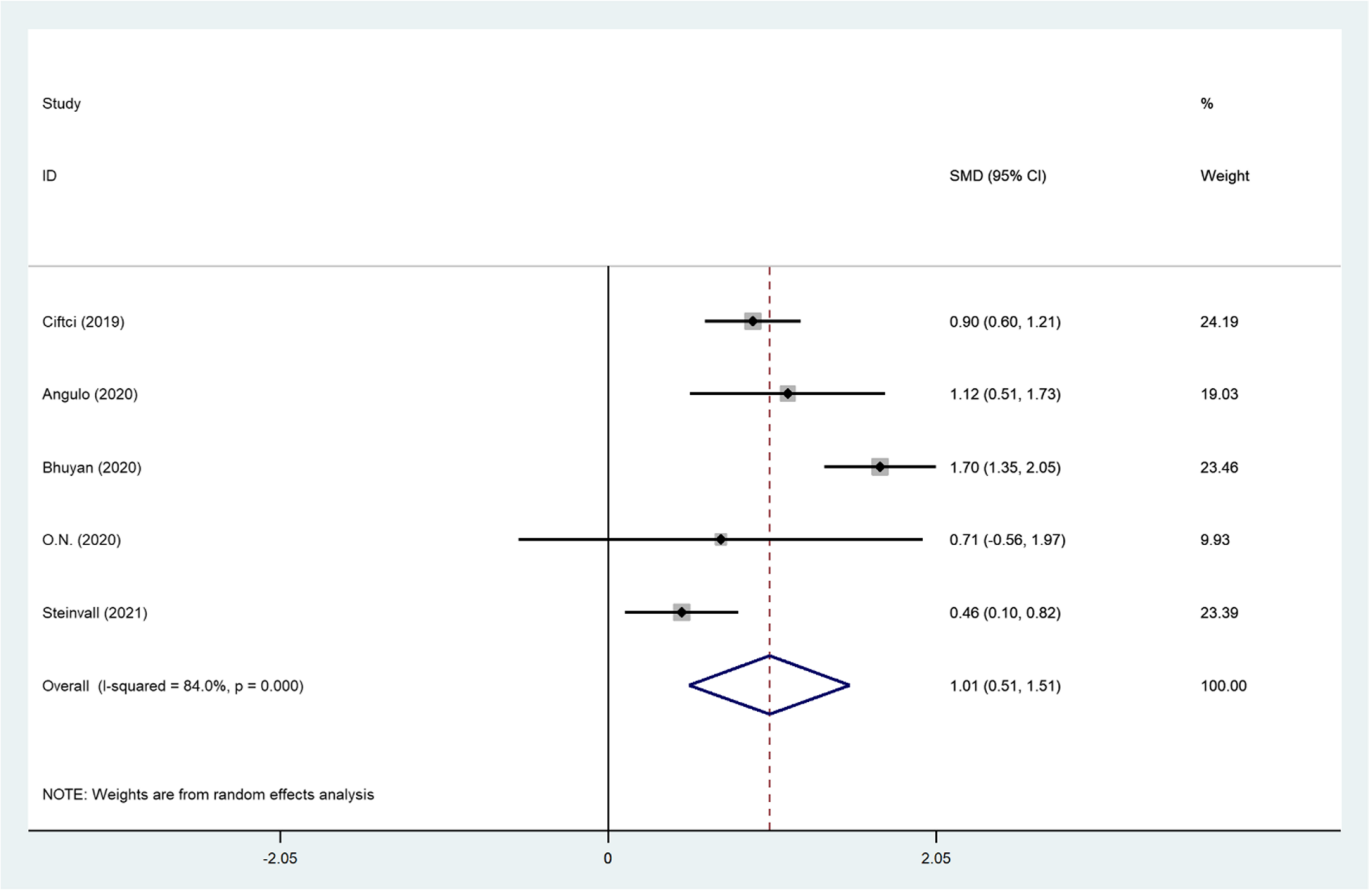
Meta-analysis of differences in NLR level of the first 24–48 h between survivor and non-survivor burned patients

To focus on the immediate postinjury-period, we analyzed the NLR data, collected in the first 24–48 h separately. We found that in the immediate postinjury-period, the NLR level was significantly higher in non-survivors (SMD = 1.01, 95% CI; 0.51–1.51, *P* < 0.001, Fig. 3).

**Fig. 4:**
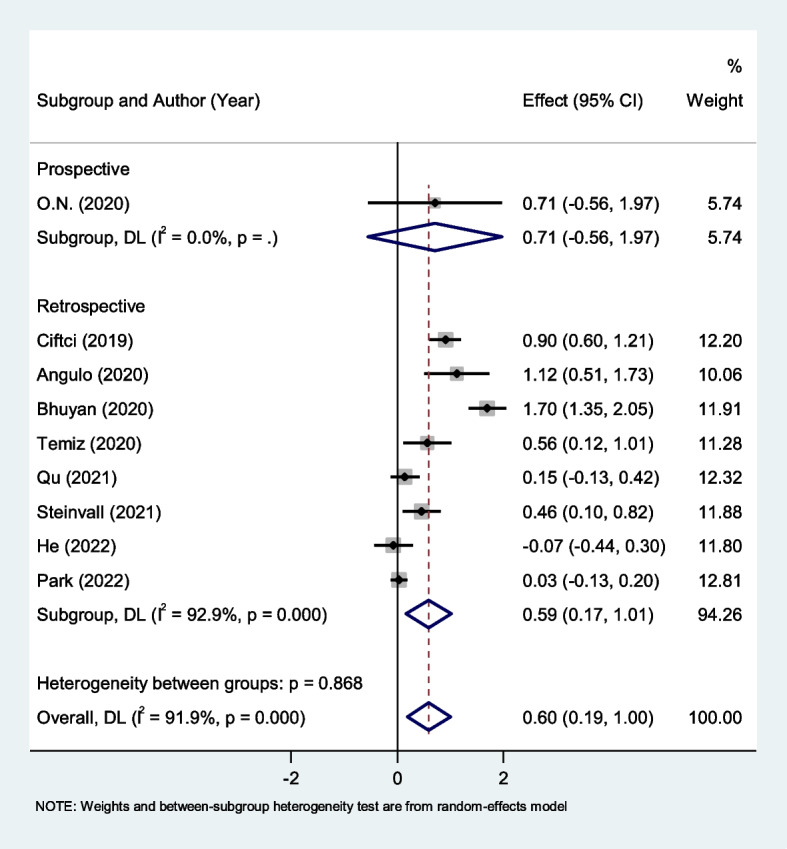
Subgroup analysis of differences in NLR level between survivor and non-survivor burned patients according to study design

Figure 4 shows the subgroup analysis according to study design. We found eight retrospective studies [43–46, 48, 49] and one small prospective study [47]. Non-survivors had elevated levels of NLR compared to survivors in the retrospective studies (SMD = 0.59, 95% CI = 0.17–1.01, *P* = 0.001). However, for the prospective studies no significant difference was observed (*P* = 0.304).

### Comparison of PLR between survivors and non-survivors

One study assessed the PLR level of patients on day of admission to the hospital [43], one study assessed it on the day before surgery [60], and one study did not report this data [49] (Tables 1 and 2).

**Fig. 5:**
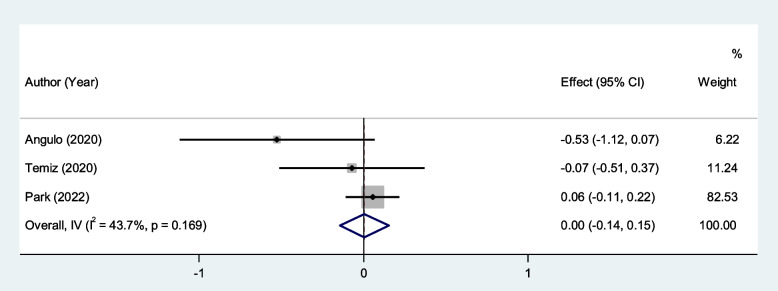
Meta-analysis of differences in PLR level between survivor and non-survivor burned patients

After pooling the data of eleven studies with 952 burned patients, including 717 survivors, we found that there was no association between PLR and mortality among burn patients (SMD = 0.00, 95% CI; -0.14–0.15, *P* < 0.001, Fig. 5). The results of the studies did not show significant heterogeneity (I^2^ = 43.7%, *P* = 0.16). We therefore used fixed effect model in our meta-analysis.

### Comparison of CRP between survivors and non-survivors

Five studies assessed the CRP level of patients at time of admission to the hospital [47, 50, 63, 65, 66], one study assessed it between post-burn day 7 and 10 [55], one reported it on Day 3 [61], one in the first 48 h after admission [62], one in less than 6 h after admission [52], and two studies did not report this data [49, 64] (Tables 1 and 2).

**Fig. 6:**
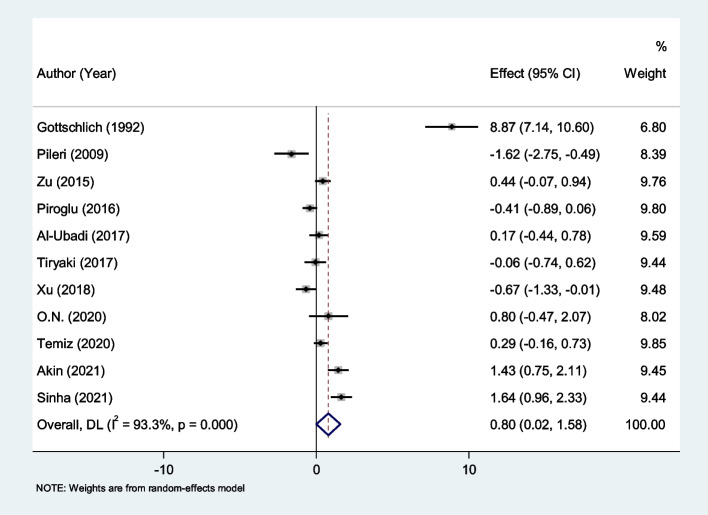
Meta-analysis of differences in CRP level between survivor and non-survivor burned patients

After pooling the data of seven studies with 709 burned patients, including 541 survivors, CRP was significantly higher in non-survivors (SMD = 0.80, 95% CI; 0.02–1.58, *P* = 0.04). The results of the studies, however, showed significant heterogeneity (I^2^ = 93.3%, *P* < 0.001; Fig. 6). We therefore used the random effect model in our meta-analysis.

**Fig. 7:**
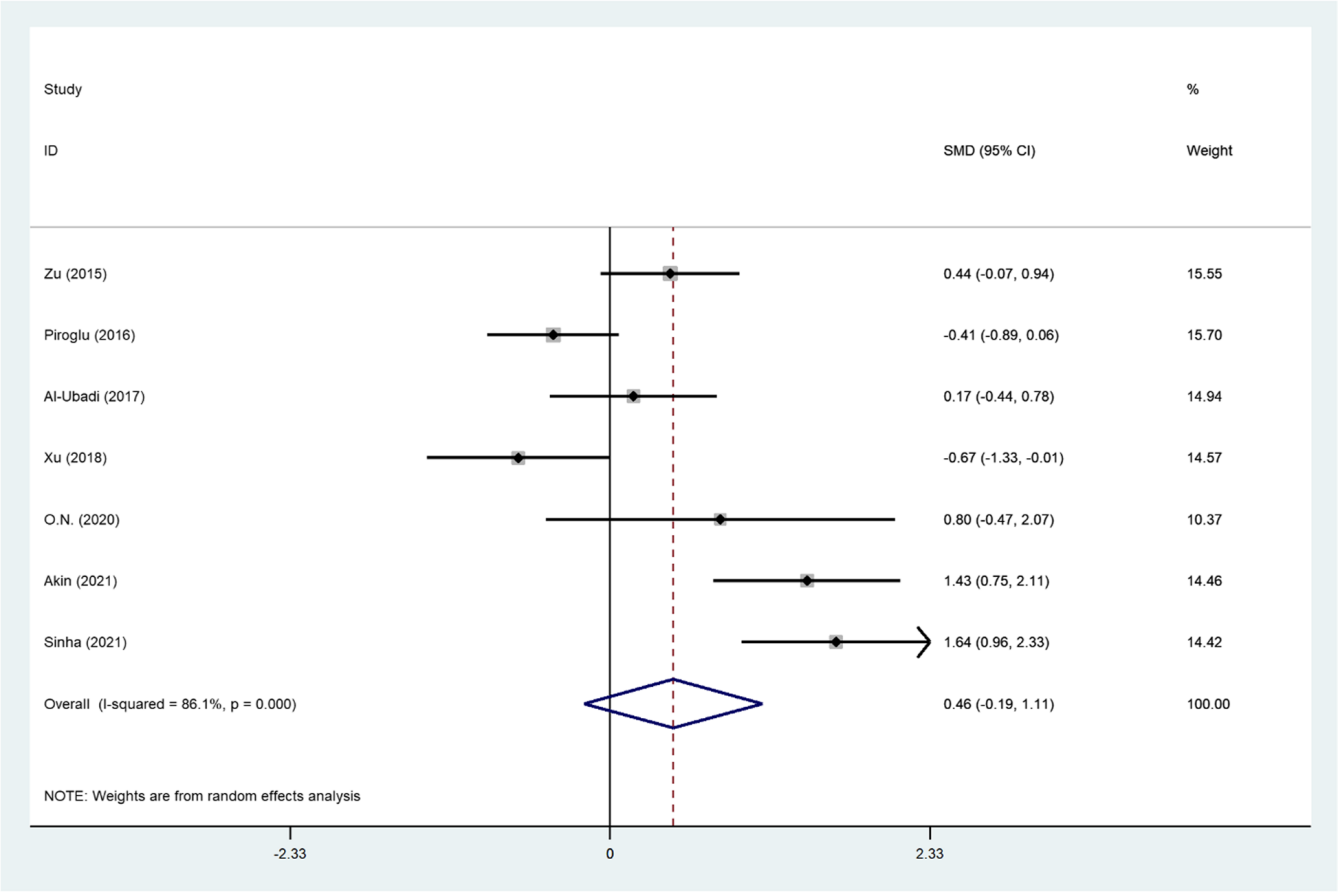
Meta-analysis of differences in CRP level of the first 24–48 h between survivor and non-survivor burned patients

However, analyzing the CRP data, collected in the first 24–48 h, showed different results; the level of this biomarker was not different between survivors and non-survivors in the immediate postinjury-period (SMD = 0.46, 95% CI; -0.19–1.11, *P* = 0.16, Fig. 7).

**Fig. 8:**
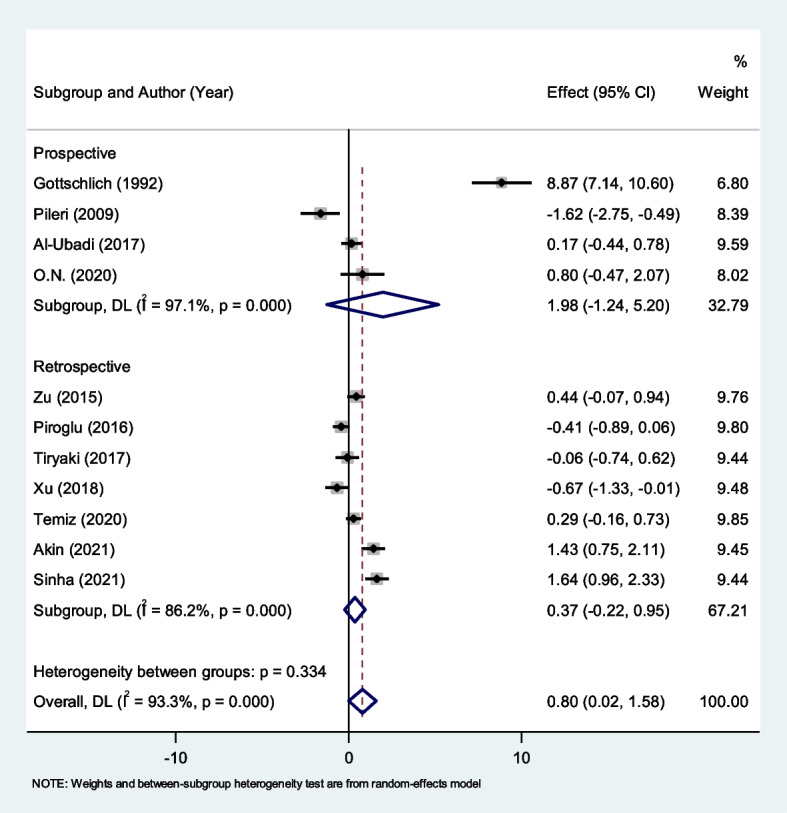
Subgroup analysis of differences in CRP level between survivor and non-survivor burned patients according to study design

As seen in Fig. 8, we found seven retrospective studies and four prospective studies in the subgroup analysis according to study design. Type of study design, retrospective (SMD = 0.37, 95% CI = -0.22–0.95, P = 0.22) or prospective studies (SMD = 1.98, 95% CI = -1.24–5.20, *P* = 0.22), showed no difference in predictive value for non-survivors verse survivors indicating generalizability (Fig. 8).

### Comparison of PCT between survivors and non-survivors

Five studies assessed PCT level of patients at time of admission to the hospital [53, 57, 63, 65, 66], one study assessed it after informed consent [54], one reported it on Day 3 [51], one in the first 48 h after admission [62], one within 48 h after injury [59], one in less than 6 h after admission [52], and one study did not report this data [58] (Tables 1 and 2).

**Fig. 9:**
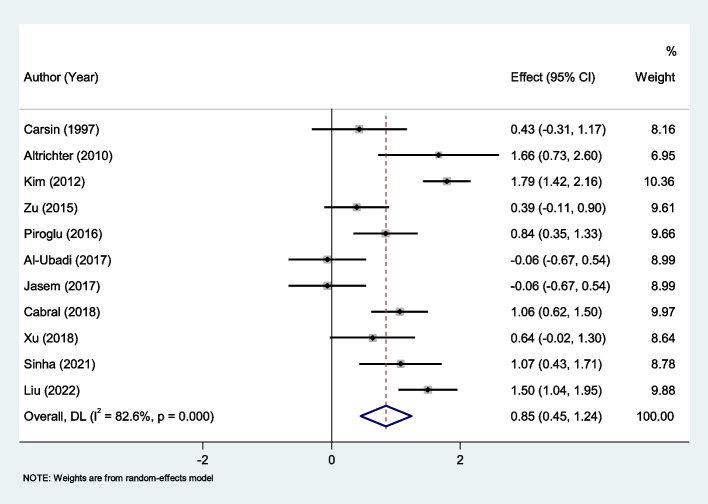
Meta-analysis of differences in PCT level between survivor and non-survivor burned patients

In this meta-analysis, we found eleven studies with 836 burned patients, including 584 survivors. The pooled results showed that PCT was significantly higher in non-survivors than survivors (SMD = 0.85, 95% CI; 0.45–1.24, *P* < 0.001). The results of the studies, however, showed significant heterogeneity (I^2^ = 82.6%, *P* < 0.001; Fig. 9). We therefore used the random effect model in our meta-analysis.

**Fig. 10:**
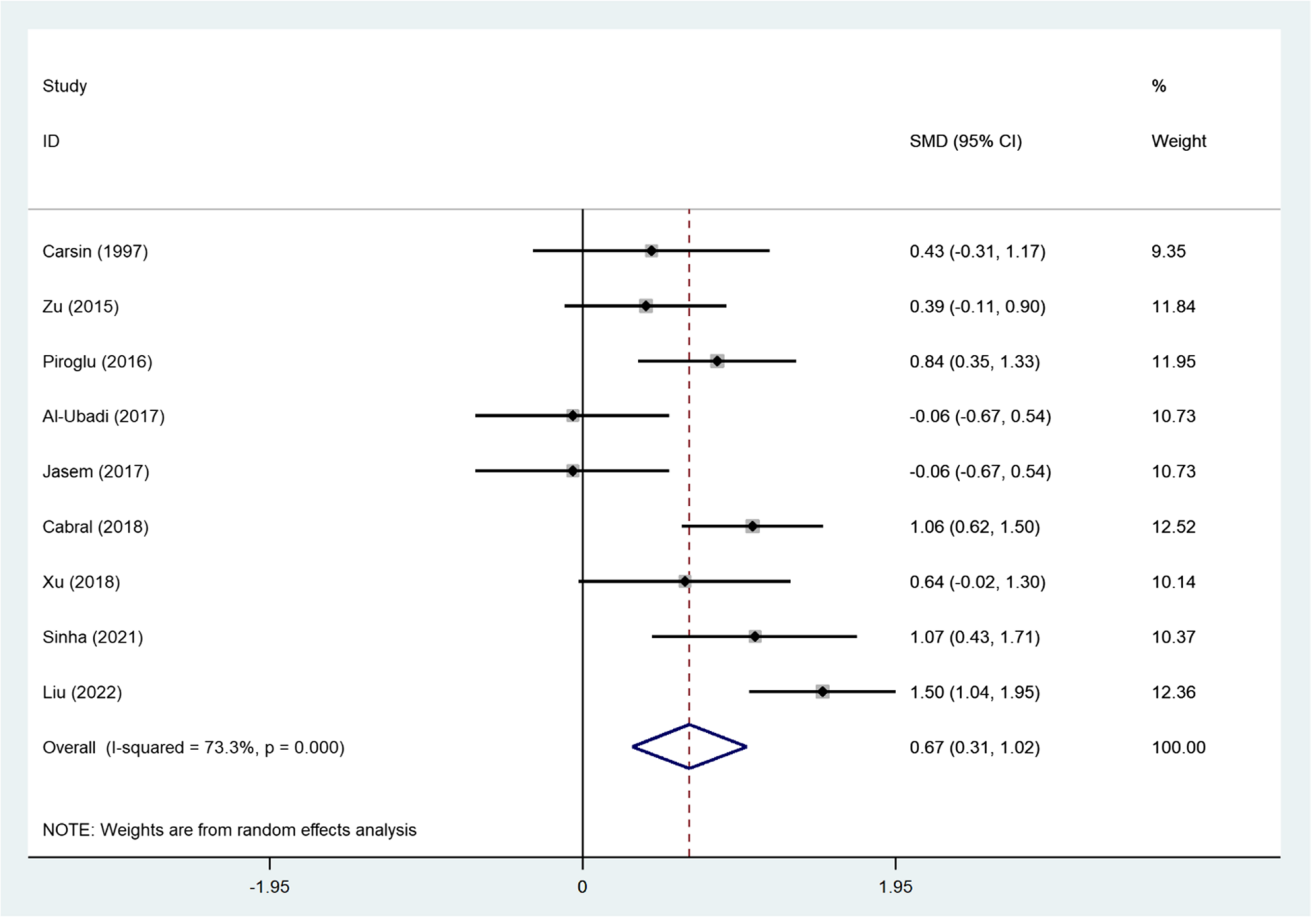
Meta-analysis of differences in PCT level of the first 24–48 h between survivor and non-survivor burned patients

When we analyzed the PCT data, collected in the first 24–48 h, we found similar results; the PCT level was significantly higher in non-survivors in the immediate postinjury-period (SMD = 0.67, 95% CI; 0.31–1.02, *P* < 0.001, Fig. 10).

**Fig. 11:**
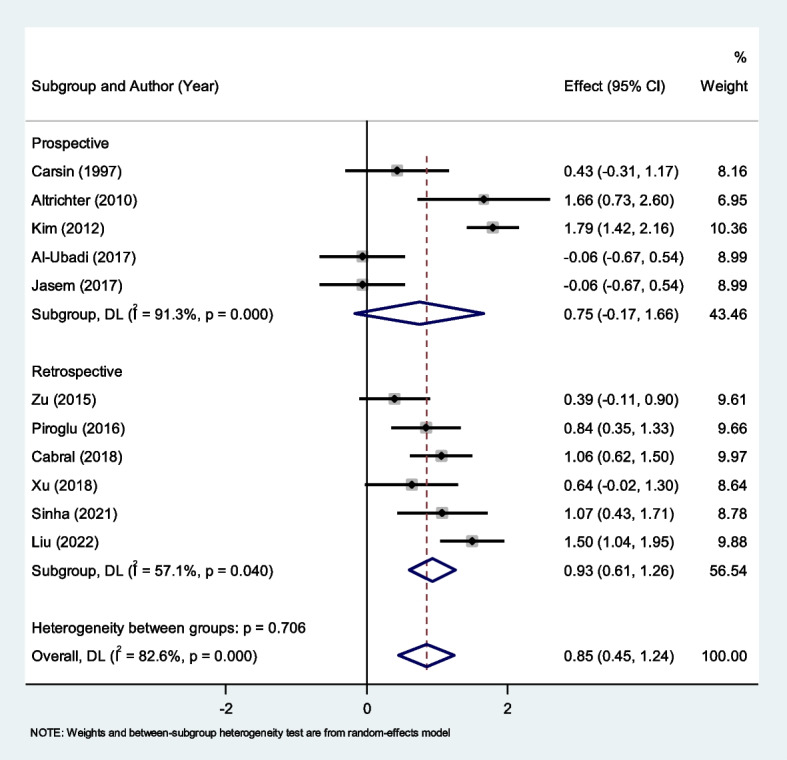
Subgroup analysis of differences in PCT level between survivor and non-survivor burned patients according to study design

We then conducted the subgroup analysis according to the study design. Pooling the results of six retrospective and five prospective studies showed that non-survivors had elevated levels of PCT compared to survivors in retrospective studies (SMD = 0.93, 95% CI = 0.61–1.26, *P* < 0.001) but not in prospective studies (SMD = 0.75, 95% CI = -0.17–1.66, *P* = 0.11) (Fig. 11).

### Publication bias

**Fig. 12:**
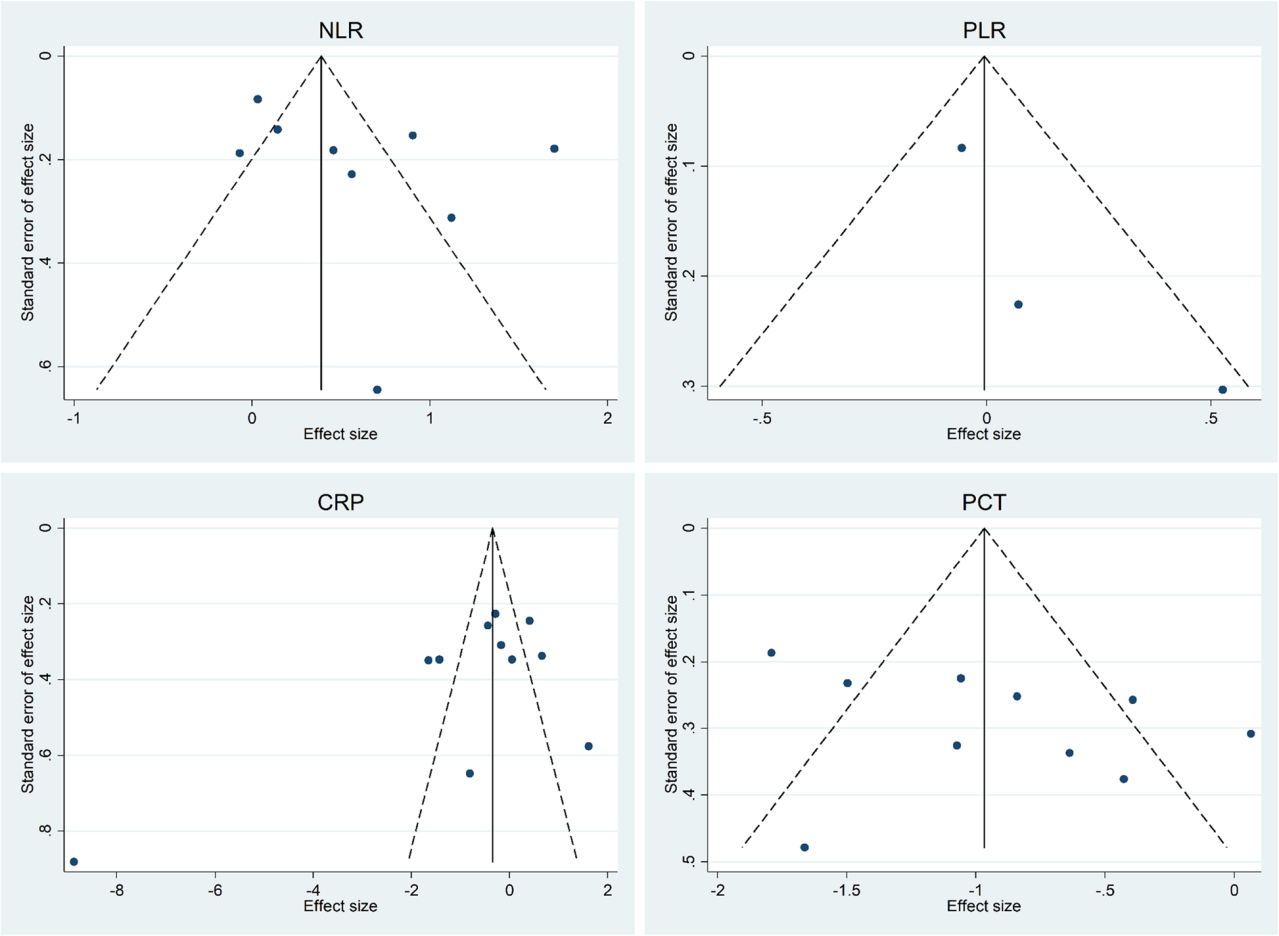
Funnel plots assessing publication bias

Figure 12 shows no publication bias among studies on the role of NLR, PLR, CRP, and PCT in detection of mortality for burn patients.

### A comparison of the accuracy of the four biomarkers

The based cut-off value for NLR was 13, with a sensitivity of 69.2% and specificity of 76%. The based cut-off value for CRP was 71, with a sensitivity of 100% and specificity of 72.22%. The based cut-off value for PCT was 1.77, with a sensitivity of 93.33% and specificity of 72.22.

## Discussion

During the early post-burn stage, cardiac, pulmonary, and renal failure ultimately results in rapid deterioration and death for those with severe burn injury [68]. Mortality in the acute setting howcer has been reduced in recent years due to effective immediate treatment [69]. Mortality remains high, however, in the late post-burn stage due to infection, sepsis, and MODS [70]. The delay in the diagnosis and treatment of such complications leads to increased mortality. An easily accessible prognostic biomarker is vital to provide earlier diagnosis and to guide treatment.

Our meta-analysis of seven studies found a significantly higher NLR in the non-survivor group of burn patients compared to the survivor group. It is essential to determine at which point the NLR between both groups becomes significant to detect change in the inflammatory process, as this would indicate when clinicians should intervene. The NLR increases with an increase in circulating neutrophils or a decrease in circulating lymphocytes and becomes more concerning as the ratio approaches or surpasses 10 [71]. Hu and colleagues found that an NLR above 14 on the admission of burn patients correlated with worse outcomes and decreased survival [72]. This study also suggested that a decreasing NLR during the first three days post-burn is associated with increased survival and appropriate treatment response. Specifically, NLRs less than 14, 13, and 7.5 on days 1, 2, and 3, respectively showed positive trend [72]. Similarly, Hwang and colleagues found an elevated NLR on admission to the emergency department to independently predict mortality in septic patients, as a high NLR indicated increased mortality risk [73].

In 2021, Qiu and colleagues followed the NLR of burn patients for 90 days. Their results suggest an elevated NLR on day three post-burn is the earliest predictor of mortality between survivor and non-survivor groups [46]. Neutrophil count illustrates the physiologic response to external stimuli or the result of injury. Data suggest that the NLR increases within 6 h in response to acute physiologic stress [3]. Hampson and colleagues mapped the course of neutrophils following a burn in their study. Neutrophil counts dramatically increased within 24 h, drop to nearly normal physiologic levels on day 3, and rise again on day 7 [74]. Neutrophils then return to physiologic levels after the first month post-burn [74]. Deveci and colleagues found that lymphocyte counts were significantly decreased by day 3, and these abnormal levels were associated with poor outcomes [75].

While neutrophilia is observed in burn patients, it is essential to consider that remaining neutrophils may have decreased function. Hampson and colleagues noted a significant decrease in neutrophils' oxidative burst capacity and phagocytotic index (PI) following a burn injury [74]. Additionally, these functions were negatively correlated with the severity of the burn injury, suggesting a more significant neutrophil dysfunction in more severe injuries. This ultimately leaves the patient more susceptible to bacterial infection and sepsis [74]. Hampson and colleagues compared burn patients with one or more septic episodes during their treatment course to burn patients without these septic episodes. They found that the decreased oxidative burst capacity remained low for seven days in both groups, though it only persisted in patients that developed sepsis [74]. As septic patients maintained a lower PI, a significant difference in PI between both groups suggests a decreased ability to phagocytose bacteria [74]. Defective opsonization has also been observed and likely contributes to the infection prevalence among burn patients, though the association is unclear [75]. Finally, the decrease in neutrophil function can largely be attributed to increased circulating immature granulocytes (IG). While both groups exhibit elevated circulating neutrophils, patients who developed sepsis maintained higher circulating IGs, specifically between 7- and 14-days post-burn [74]. The observed oxidative burst capacity and PI are most reduced during this critical period. Therefore, while an inflammatory surge is noted, the function of the neutrophils is vastly decreased. This increases the infection risk of severely burned patients. These findings support using the NLR as a prognostic and diagnostic tool in treating sepsis in patients with severe burns.

An increased NLR may also be a risk factor for MODS, though the literature primarily focuses on acute kidney injury (AKI). Tissue destruction may include structures below the skin and infection within the visceral organs [76]. Karakaya and colleagues found that burn patients who maintained an increased NLR also exhibited a higher incidence of AKI. This is likely due to the impact concomitant infections have on AKI pathogenesis [76]. Interestingly, Younan and colleagues found an increased NLR to be significantly associated with both the development of organ failure. This was particularly important for the number of organs that fail in male trauma patients but not female trauma patients [77]. Further research into the potential role of sex hormones on inflammatory cells could provide greater clarity in guiding treatment for patients with severe burns.

Additionally, our meta-analysis of 11 CRP and 11 PCT studies found a significantly higher CRP and PCT in the non-survivor burn patients compared to the survivor group. Understanding the levels at which the CRP and PCT between both groups become significant in the inflammatory process is vital, as this would further indicate when interventions need to be started. While CRP and PCT remain the most widely used biomarkers in patients with sepsis, level of change and time course for associated mortality have yet to be fully elucidated [78].

Generally, CRP levels taken from patients with burn injuries are consistently high for the duration of their hospitalization, and changes in CRP are not reliable [79]. Yigit and Yigit found elevated CRP was not directly associated with septic burns. They found no correlation between patient clinical status and CRP levels [79]. Their study concluded that CRP is useful as a biomarker regarding inflammation, but its efficacy as a marker for predicting sepsis course remains uncertain [79]. Interestingly, Ticinesi and colleagues found elevated CRP levels on admission are helpful for detecting sepsis in elderly patients, but not necessarily younger patients [16]. Through their meta-analysis, Tan and colleagues found that CRP's diagnostic role for sepsis is significantly less accurate and less specific than PCT [80]. Our results showed that CRP was most accurate for determining mortality but does not necessarily delineate contributing cause.

PCT, a precursor protein for calcitonin, is mainly synthesized by thyroid C-cells. In individuals without systemic inflammation, serum PCT is undetectable because the protein is not released into the bloodstream under normal conditions [81–83]. If bacterial infections lead to sepsis, PCT synthesis is activated in nearly all tissues, making it identifiable in the bloodstream. Bacterial toxins, including endotoxin and cytokines like tumor necrosis factor-alpha, interleukin (IL)-1beta, and IL-6, stimulate the synthesis of PCT in such cases. [84] Most viral infections do not stimulate PCT synthesis because cytokines generated during viral infections block TNF-alpha production [81–83]. In addition, PCT has a long half-life, a broad biological range, and a quick induction period upon bacterial stimulation [85]. Consequently, PCT proves to be a valuable tool with excellent discriminatory properties for distinguishing between bacterial and viral inflammations, offering prompt and readily available results [86]. Monitoring PCT levels in burned patients is essential due to the increased susceptibility to infections, particularly nosocomial ones. Elevated PCT levels indicate a systemic response to bacterial invasion, thus proving to be a valuable marker for bacterial infections in this patient group. In sepsis, a significant rise in PCT levels may occur due to immune response dysregulation, and its sustained elevation could signify a more severe and prolonged inflammatory state. This sustained elevation in PCT levels can substantially impact the overall prognosis of burn patients [87–90].

Many studies have validated the significance of elevated PCT levels in the diagnosis of burn sepsis in the setting of infection, the most common complication and cause of death in burn patients [21, 88, 91–93]. Piroglu and colleagues suggested that the risk of mortality in patients with PCT levels above three ng/mL versus those below three ng/mL was 21.3 times higher, and the diagnostic value of PCT was greatest with levels above three ng/mL [62]. Elevated PCT in the early phase of extensive burn patients is most closely correlated with APACHE-II score, degree of inhalation, and burn index. As mentioned previously, Xu and colleagues confirmed these correlations and evaluated the value of PCT in the early stages of the disease. Their results suggest that an early-phase PCT greater than 4.275 ng/mL was a significant risk factor for sepsis within 60 days following extensive burns [28].

PCT levels can help clinicians differentiate between systemic inflammation and sepsis in cases where infection is suspected, and the response to treatment can be monitored using PCT, blood cultures, and clinical assessment. Yigit and Yigit found that PCT levels in patients returned to their baseline values as they improved clinically [79]. Subsequently, PCT levels remained consistently elevated in patients who progressed to develop burn sepsis [79]. So, PCT is not directly related with inflammation as CRP and, for burn patients, its kinetics help clinicians to better distinguish a systemic inflammatory state from septic episodes. Even the small PCT increases immediately after burn injury or surgical interventions are predictable and, if infection is not present, will rapidly subside.

Finally, our meta-analysis of three PLR studies found no association between PLR and mortality among burn patients. Literature suggests the roles of both platelets and lymphocytes in the inflammatory process. Platelets induce the release of inflammatory cytokines and interact with various bacteria and immune cells [94–97]. Decreased lymphocytes suggest suppressed immune and inflammatory responses [98–100]. Therefore, the PLR could be considered a systemic inflammatory biomarker. While many diseases may rely on the PLR (i.e., acute kidney injury [101], hepatocellular carcinoma [102], myocardial infarction [103], non-small cell lung cancer [104], rheumatoid arthritis, and systemic lupus erythematosus), the literature remains unclear whether or not PLR is reliable in predicting sepsis.

Shen and colleagues found that a high PLR on admission is significantly associated with increased sepsis mortality, though only in the setting of vasopressor use and acute kidney injury [41]. Orak and colleagues found a higher PLR in patients with sepsis who did not survive when compared to surviving patients [40]. Djordjevic and colleagues found higher PLR values to predict mortality in trauma patients but no predictive value in sepsis, peritonitis, or pancreatitis [39]. In their study, Hou and colleagues suggest using PLR in predicting early sepsis with values greater than 210 showing high risk [42]. Further research is ultimately needed to understand the reproducibility and validity of PLR in predicting sepsis, particularly when compared to the previously evaluated biomarkers.

### Strengths and limitations

This report, however, has at least four limitations. The first limitation of the data extracted from the relevant articles was that they did not permit an assessment of the relationship between ratios and burn severity. As a result of differences between burn severity, treatment regimens, center protocols, study populations, and times of blood tests used to calculate biomarkers, heterogeneity was more significant than expected. Therefore, we must conduct more extensive prospective studies to examine general validity. Thirdly, several studies have biases in their selection or publication, which must be considered. Fourthly, other biomarkers of immune function, such as cytokines, were not assessed; thus, it is impossible to determine if elevated NLR represents an independent marker of immune system abnormalities. In addition, based on the fact that in severe burn patients a systemic inflammatory state is always present, isolated levels of all biomarkers must be taken with caution and the analysis of its kinetics is much more reliable. Nonetheless, there were three main strengths in the present review. First, the present study, to our best knowledge, serves as the first meta-analysis exploring the correlation between NLR and survival in burn patients. Second, the studies were included in the final analysis based on explicit inclusion and exclusion criteria. Third, our systematic search—in conjunction with a manual review of references from resulting articles without any limitation on language or date- has ensured a thorough and reliable examination of literature and is a notable strength of this study.

## Conclusion

Inflammation is strongly associated with NLR, PCT, and CRP levels, which can be used to predict the severity of an inflammatory process like burns. Although all three of them had high sensitivity and specificity, CRP is the best biomarker for predicting mortality among burn patients, based on the sum value of sensitivity and specificity, but does not clearly delineate sepsis course. PCT is obviously a marker of sepsis, so its elevated level is presumably associated with a higher incidence and severity of sepsis among non-survivors. Knowing that sepsis is the main cause of mortality in burns, the association is not surprising, but it cannot be used as a prognosis estimator when late data are available. A high biomarker value implies a more severe inflammatory response. Clinical worsening, a worse prognosis, and mortality could result from more severe inflammation. Our study indicated that the levels of these biomarkers among burned non-survivors are significantly higher than survivors, especially for CRP. NLR and PCT have potential role in determining sepsis development and monitoring treatment response. The markers are low-cost and can serve as potential clinical predictors that can be employed even in resource-constrained settings. However, a sequential determination of a series of biomarkers is better than just one value for predicting the prognosis among burn patients. Combination mapping over time with prognostic studies is warranted in terms of both prognostication and treatment response.

## Data Availability

The dataset supporting the conclusions of this article is included within the article.

## Abbreviations

MODS: Multiple organ dysfunction syndromes
NLR: Neutrophil to lymphocyte ratio
CRP: C-reactive protein
PCT: Procalcitonin
PLR: Platelet to lymphocyte ratio
PRISMA: Preferred Reporting Items for Systematic Reviews and Meta-Analyses
SD: Standard deviation
IQR: Interquartile range
OR: Odds ratio
HR: Hazard ratio
RR: Risk ratio
NOS: Newcastle-Ottawa Quality Assessment Scale
SMD: Standard mean difference
CI: Confidence interval
PI: Phagocytotic index
IG: Immature granulocytes
AKI: Acute kidney injury
